# Optimising Survey Strategies for the Endangered Romer's Tree Frog (*Liuixalus romeri*): Effects of Diel, Seasonal and Weather Variation on Acoustic Detection

**DOI:** 10.1002/ece3.74491

**Published:** 2026-10-14

**Authors:** Yik‐Hei Sung, Amy Wing Lam Fok, Hiu Ching Yuen, Franco Ka Wah Leung, Michael Wai‐neng Lau, Jia Huan Liew, Simon Yung Wa Sin, Jonathan J. Fong

**Affiliations:** ^1^ School of Health, Sciences and Society University of Suffolk Ipswich UK; ^2^ Division of Science, Wu Jieh Yee School of Interdisciplinary Studies Lingnan University Hong Kong SAR China; ^3^ College of Science and Engineering James Cook University Townsville Queensland Australia; ^4^ Hong Kong Wetlands Conservation Association Hong Kong SAR China; ^5^ School of Biological Sciences The University of Hong Kong Hong Kong SAR China; ^6^ School of Natural Sciences University of Tasmania Hobart Tasmania Australia

**Keywords:** amphibian conservation, bioacoustics, detection probability, Hong Kong, passive acoustic monitoring, rainfall

## Abstract

Accurate estimation of species distribution and abundance depends on detection probability, which can vary with environmental conditions. For amphibians, calling activity and detectability are strongly influenced by diel, seasonal and weather conditions. However, these relationships remain poorly quantified for many species, limiting the optimisation of survey planning. We used passive acoustic monitoring (PAM) to investigate environmental factors of call detection in the endangered Romer's tree frog ( *Liuixalus romeri* ) in Hong Kong. Acoustic data were collected continuously across multiple sites to quantify the effects of time of day, season and weather variables on detection probability. We further compared PAM detections with active call surveys to assess concordance between methods. Detection probability exhibited strong diel and seasonal patterns, peaking during the wet season (June–August) and at night, with highest detection probability during early evening (20:00–22:00) and a secondary peak before dawn (03:00–04:00). Rainfall effects varied across the diel cycle, with positive effects during peak calling periods but negative or neutral effects at other times. Detection probability showed a non‐linear relationship with temperature, peaking at approximately 27.5°C, and declined with increasing wind speed. Agreement between PAM and active call surveys was moderate (Cohen's Kappa = 0.47), with concordant call agreement for 73% of survey nights. This indicates that the two methods are complementary rather than interchangeable. Based on these findings, we recommend conducting manual surveys during peak activity periods (June–August, 20:00–22:00), under conditions of low wind and following recent rainfall. PAM provides a valuable tool for continuous monitoring and detecting temporal changes in call detection, while manual surveys remain important for estimating abundance. Integrating both approaches can improve monitoring protocols for *L. romeri* and other amphibians.

## Graphical abstract

Accurate amphibian monitoring requires understanding how detection probability varies with environmental conditions, yet these relationships remain poorly quantified for many threatened species. Using passive acoustic monitoring, we found that call detection in the endangered Romer's tree frog ( *Liuixalus romeri* ) was strongly influenced by time of day, season, rainfall, temperature and wind speed, with peak detection during the wet season and early evening.

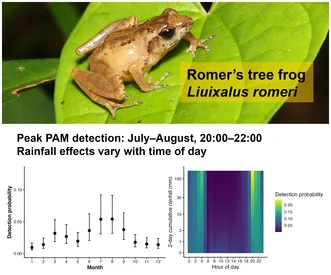

## 1 Introduction

Monitoring distribution and abundance is fundamental to the management and conservation of endangered species (Moussy et al. 2022). However, detection probability varies among taxonomic groups, with particular challenges for reliable assessment for species that are cryptic or whose activity is sensitive to environmental conditions, such as amphibians and reptiles (Baumgardt et al. 2021; Durso et al. 2011).

Amphibian surveys largely depend on the detection of calling activity, which is highly sensitive to environmental conditions. Previous studies have found that calling activity is strongly influenced by seasonality, diel cycles and weather variables such as rainfall, temperature, wind and moon phase (Leung et al. 2025; Oseen and Wassersug 2002; Ospina et al. 2013; Thompson et al. 2022). Consequently, surveys conducted under suboptimal conditions may yield inaccurate results, leading to biassed inferences and ineffective conservation actions. These environmental influences on calling activities highlight the importance of identifying appropriate environmental conditions for planning and implementing amphibian surveys and monitoring (Rush et al. 2025).

Romer's tree frog, *Liuixalus romeri* (Smith 1953), is an Endangered species with a highly restricted global distribution (IUCN 2024), inhabiting several islands in and near Hong Kong, China (Agriculture Fisheries and Conservation Department 2022). In the early 1990s, populations were translocated in response to habitat loss caused by the construction of the Hong Kong International Airport (Banks et al. 2008). Although populations have remained relatively stable since then, emerging threats—including the introduction of the non‐native greenhouse frog ( *Eleutherodactylus planirostris* ) and the use of mosquito larvicides—may pose increasing risks through competition and potential toxicity (Agriculture Fisheries and Conservation Department 2022; Lee et al. 2016). Effective population monitoring is therefore essential to detect changes in population status and evaluate these threats.

While it is well known that *L. romeri* is nocturnal and breeds during the wet season (Lau 1998), the precise diel and seasonal patterns of calling activity, and their relationships with environmental conditions, remain poorly quantified. Such information is critical for optimising survey design and improving detection probability (Steelman and Dorcas 2010). To date, surveys of this species have relied primarily on active surveys, which sample only for limited durations and may overlook variation in calling activity across time and environmental conditions.

Passive acoustic monitoring (PAM) has been widely applied in ecological and conservation research (Darras et al. 2025; Sugai et al. 2019), particularly in studies on amphibians. It offers a promising complementary approach to active surveys (Boullhesen et al. 2021; Hoefer et al. 2023; Lassandro et al. 2025). Application of PAM enables continuous, high‐resolution recording of calling data to provide information on the relationships between calling activity, diel and seasonal patterns and weather conditions.

In this study, we deployed PAM to investigate how detection of *L. romeri* calls varies across diel and seasonal scales, as well as in relation to key weather variables. We further assessed the concordance between PAM and active call surveys in detecting the species. By integrating these approaches, we aim to provide recommendations for optimising survey conditions and improving monitoring protocols for *L. romeri* , as well as provide a methodology to evaluate other amphibian species.

## 2 Methods

### 2.1 Study Species and Site

*Liuixalus romeri* is the smallest native amphibian in Hong Kong, with a snout–vent length of about 1.5 cm in males and up to 2.5 cm in females (AmphibiaWeb 2022). It has a tan brown dorsum with a dark, X‐shaped marking behind the eyes, expanded digital discs, and, in males, a single external subgular vocal sac (Chan et al. 2005). The species is nocturnal and occurs in leaf litter and understorey vegetation in woodland, forest and plantations, feeding on small arthropods (Lau 1998). Breeding occurs in shaded, slow‐flowing or standing water that is free of fish, where males produce short bursts of high‐pitched, cricket‐like advertisement calls (Chan et al. 2005). Females lay up to about 120 eggs, and larvae metamorphose within 4 to 6 weeks.

Hong Kong (22°090–22°370 N, 113°500–114°300 E) has a subtropical monsoon climate, characterised by hot, wet summers (May to September) and cooler, drier winters (November to February), with two transitional seasons in between (Dudgeon and Corlett 2004). The breeding of *L. romeri* spans from February to September and is mainly associated with the wet summer months (Lau 1998).

This study was conducted on Lantau Island and Chek Lap Kok, Hong Kong Special Administrative Region, China, where *L. romeri* naturally occurs (Chan et al. 2005; Lau 1998). We selected seven sites known to support breeding populations based on previous surveys (Figure 1).

**FIGURE 1:**
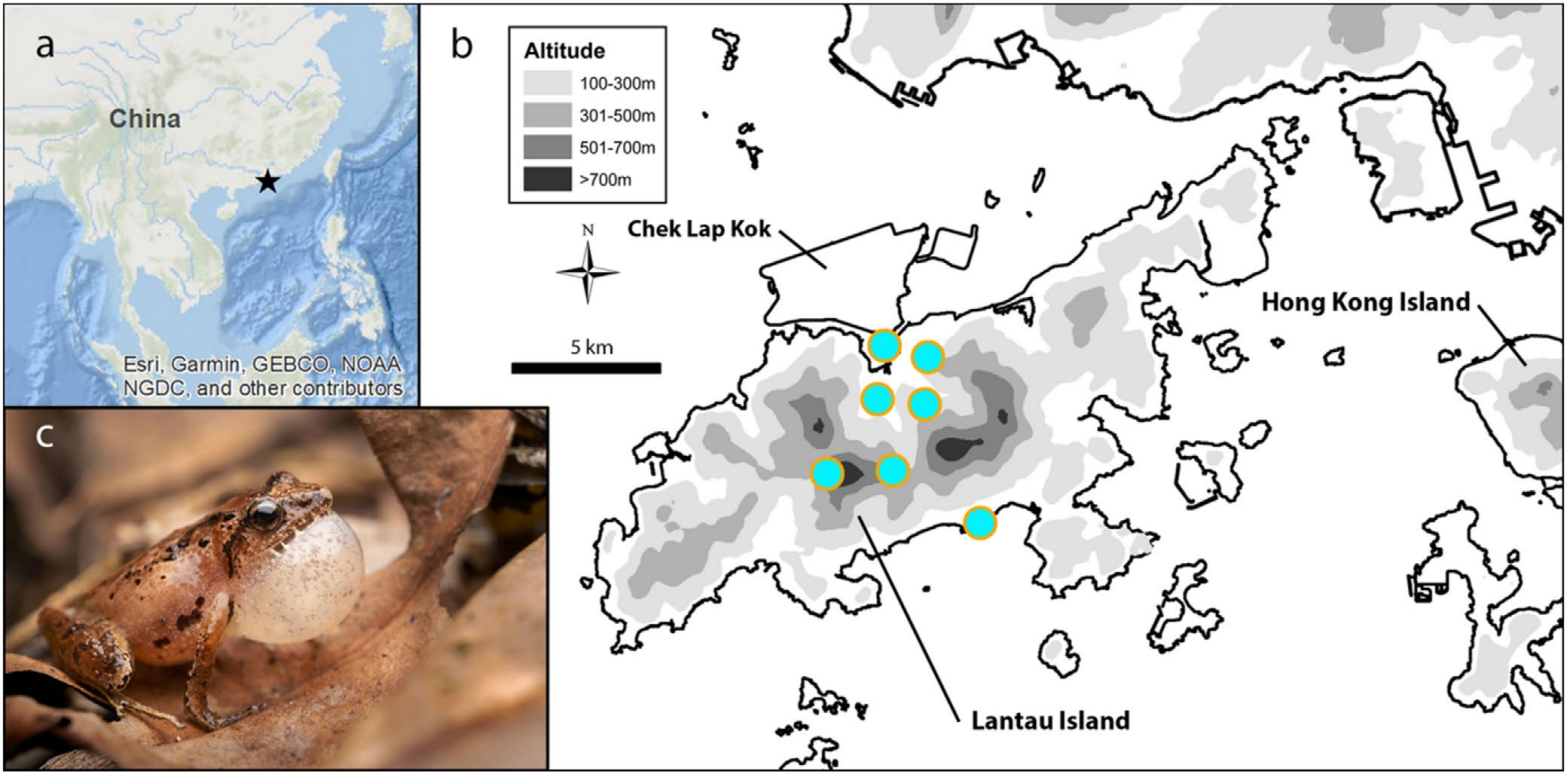
Study area and study species. (a) Location of Hong Kong in southern China, indicated by a star. (b) Seven study locations on Lantau Island and Chek Lap Kok, Hong Kong, indicated by blue dots. (c) A calling male of the study species, *Liuixalus romeri* . Photographs of *L. romeri* were taken by Daphne Wong and James Kwok.

### 2.2 Passive Acoustic Monitoring and Call Recognition

At each site, two automated recording units (AudioMoth; Open Acoustic Devices) were deployed, with paired recorders separated by 70–626 m (mean ± SD = 319 ± 236 m). At two sites, paired recorders were separated by < 100 m because of the availability of breeding sites, whereas the remaining five sites had separation distances > 100 m. Although these distances cannot guarantee complete acoustic independence, previous studies have reported shorter maximum detection ranges for some frog species, for example, approximately 50 m for *Scinax fuscomarginatus* (de Souza et al. 2020). Furthermore, a recent review reported that eight studies used recorders separated by ≤ 100 m (Lassandro et al. 2025). During our field surveys, calls of *L. romeri* were barely audible at 70 m, which suggests that acoustic overlap is limited. Although acoustic overlap could not be completely excluded, the sensitivity analysis using one recorder per site produced qualitatively similar results and the conclusions remained unchanged (Appendix S1). We therefore retained both recorders of each site in the main analysis to maximise spatial sampling within sites, while recognising that the observations may not be fully acoustically independent. Recorders were mounted on trees approximately 1–1.5 m above ground, and operated continuously from June 2023 to March 2025. Recordings were scheduled for 5 min per hour across a full 24‐h cycle. Audio files were stored in WAV format (sampling rate: 48 kHz; bit‐depth: 16; medium gain).

Call detection was conducted using the ARBIMON web‐based platform (Aide et al. 2013), applying a template‐based classifier. Templates were constructed from high signal‐to‐noise recordings without overlap from non‐target organisms by manually selecting representative calls using the visualiser on ARBIMON. Spectrograms were generated using a short‐time Fourier transform (STFT) with a 512‐point FFT, a Hann window, and 50% overlap. Regions of interest were defined to include a four‐note sequence (0.48 s duration; 4.7–7.6 kHz), which represents a subsection of the longer advertisement call type of *L. romeri* . We manually assessed 1195 recordings collected from 10 sites across multiple days to evaluate the classifier's performance. Precision and recall were calculated under different detection thresholds. At a threshold of 0.2, precision was 0.64 and recall was 0.83, whereas at a threshold of 0.3, precision increased to 0.89 but recall decreased to 0.34. The 0.2 threshold had a higher F1 score than the 0.3 threshold (0.72 vs. 0.49). Given the trade‐off between false positives and false negatives, a threshold of 0.3 was adopted for the main analysis to prioritise high‐confidence detections. We acknowledge that the low recall may bias detections toward periods of higher calling intensity and may lead to underestimation of overall detection probability.

### 2.3 Active Call Surveys

Active call surveys were conducted at all study sites monthly between August 2023 and September 2024. Surveys consisted of 5‐min point counts conducted at least 1 h after sunset with a 5‐min acclimatisation period before each count. At each site, two survey points 5–10 m from the recorder locations were sampled. During each survey, observers recorded the number of calling individuals detected. To evaluate the concordance between PAM and active call surveys, we quantified agreement in detection outcomes at the level of Individual 5‐min survey periods.

### 2.4 Environmental Factors

We selected seven environmental factors based on their known impacts on frog calling activity and data availability—hours of day, months, rainfall, air temperature, humidity, wind speed and moon phase. Daily weather data, including mean temperature, total rainfall, mean humidity and mean wind speed, were obtained from the nearest Hong Kong Observatory weather station at Hong Kong International Airport (Hong Kong Observatory 2026). Rainfall was calculated as the sum of precipitation on the sampling day and the preceding day (2‐day cumulative rainfall) to account for lagged rainfall effects.

### 2.5 Statistical Analysis

Acoustic data were aggregated to hourly sampling units. Each 5‐min recording was divided into five 1‐min segments for analysis in ARBIMON. For each hour, we calculated a binary detection variable indicating whether at least one call was detected, and a calling index defined as the number of 1‐min segments (out of five) detecting calls. Hours with incomplete sampling (i.e., < 5 recordings) were excluded from the analysis.

To investigate the relationship between detection and calling index with environmental factors, we used generalised linear mixed models (GLMMs) implemented in the *glmmTMB* package (Brooks et al. 2017). Detection probability was modelled using a binomial error distribution, while calling index was modelled using a negative binomial distribution (Zuur et al. 2009). Fixed factors included hour of day, month, rainfall, temperature, humidity, wind speed, moon phase and temporal autocorrelation represented by the lagged response variable (Zuur et al. 2010). We also included an interaction between rainfall and hour of day because rainfall may affect calling activity patterns. We included site and date as random factors to account for spatial and temporal dependence. Rainfall was transformed using a log function (Zuur et al. 2009). Diel cycle and moon phase were modelled using sine and cosine terms to account for cyclic patterns (Stolwijk et al. 1999). To account for potential non‐linear relationships, rainfall was included as both linear and quadratic terms to capture potential non‐linear effects, while temperature was modelled using natural cubic splines. All continuous environmental factors were standardised before modelling. We tested for multicollinearity among fixed factors using adjusted generalised variance inflation factors (GVIFs; Fox and Monette 1992). Adjusted GVIFs were below 1.6 except for the linear and quadratic terms of rainfall, with GVIFs of 3.5 and 3.7, respectively; all fixed effects were retained in the models. Model diagnostics were conducted using the *DHARMa* package (Hartig 2026).

To visualise interactions between rainfall and time of day, we fitted an additional model in which sine and cosine terms for hour of day were replaced with categorical hour of day, allowing visualisation of hour‐specific activity patterns of the rainfall effect.

To compare concordance between PAM and active call surveys, we evaluated PAM detections under two temporal sampling windows: (1) the exact hour corresponding to the active call survey; and (2) detection across the full night (19:00 to 07:00). Agreement between methods was quantified using a contingency table and Cohen's kappa coefficient (Cohen 1960). We also compared naïve detection probability between the two methods, estimated as the proportion of paired survey nights on which the species was detected. We calculated Wilson 95% confidence intervals and compared the two methods using McNemar's tests (Brown et al. 2001; McNemar 1947). All analyses were conducted in R software (R Core Team 2024).

## 3 Results

A total of 834,180 1‐min recordings (in 166,836 h) were analysed using automated PAM detection. *Liuixalus romeri* calls were detected in 15,943 recordings and 9722 h. Detection probability varied strongly with hour of day, rainfall, season, wind speed and temperature (Table 1). The cosine component of hour of day was strongly positive (*β* = 0.580, *p* < 0.001), indicating a pronounced peak in detection during specific periods of the daily cycle, whereas the sine component was not significant (*p* = 0.117). Model predictions revealed a clear diel pattern, with detection probability highest at night and low during the day (06:00–18:00; Figure 2a).

**TABLE 1:** Fixed factors included in generalised linear mixed models (GLMMs) used to model detection probability of *Liuixalus romeri* in Hong Kong (2023–2025). Fixed factors include diel cycle, rainfall, season (month), weather variables, moon phase and temporal autocorrelation. For season, January was used as the reference category. Significant effects (*p* < 0.05) are shown in bold. Conditional R^2^: 0.439; marginal R^2^: 0.251.

Factor category	Fixed factor	Estimate	SE	*z*	*p*
Diel cycle	Sine (hour)	−0.036	0.023	−1.569	0.117
	**Cosine (hour)**	**0.580**	**0.024**	**24.188**	**< 0.001**
Rainfall	**Log (rainfall)**	**−0.154**	**0.059**	**−2.596**	**0.009**
	Log (rainfall)^2^	0.000	0.014	0.027	0.979
Season	**February**	**0.394**	**0.136**	**2.905**	**0.004**
	**March**	**1.228**	**0.139**	**8.820**	**< 0.001**
	**April**	**1.054**	**0.181**	**5.829**	**< 0.001**
	**May**	**0.723**	**0.185**	**3.910**	**< 0.001**
	**June**	**1.360**	**0.209**	**6.517**	**< 0.001**
	**July**	**1.778**	**0.211**	**8.428**	**< 0.001**
	**August**	**1.787**	**0.199**	**8.959**	**< 0.001**
	**September**	**1.401**	**0.194**	**7.220**	**< 0.001**
	**October**	**0.628**	**0.165**	**3.817**	**< 0.001**
	**November**	**0.455**	**0.139**	**3.276**	**0.001**
	**December**	**0.389**	**0.126**	**3.074**	**0.002**
Weather	**Temperature (spline 1)**	**1.189**	**0.216**	**5.495**	**< 0.001**
	**Temperature (spline 2)**	**1.366**	**0.615**	**2.220**	**0.026**
	Temperature (spline 3)	0.434	0.243	1.791	0.073
	Humidity	−0.002	0.038	−0.043	0.966
	**Wind speed**	**−0.090**	**0.024**	**−3.671**	**< 0.001**
Moon phase	Sin (moon phase)	0.063	0.033	1.895	0.058
	Cosine (moon phase)	0.022	0.033	0.667	0.505
Autocorrelation	**Lagged detection**	**2.470**	**0.029**	**85.598**	**< 0.001**
Interactions	**Log (rainfall) × sine (hour)**	**−0.068**	**0.013**	**−5.325**	**< 0.001**
	**Log (rainfall) × cosine (hour)**	**0.261**	**0.016**	**16.270**	**< 0.001**

**FIGURE 2:**
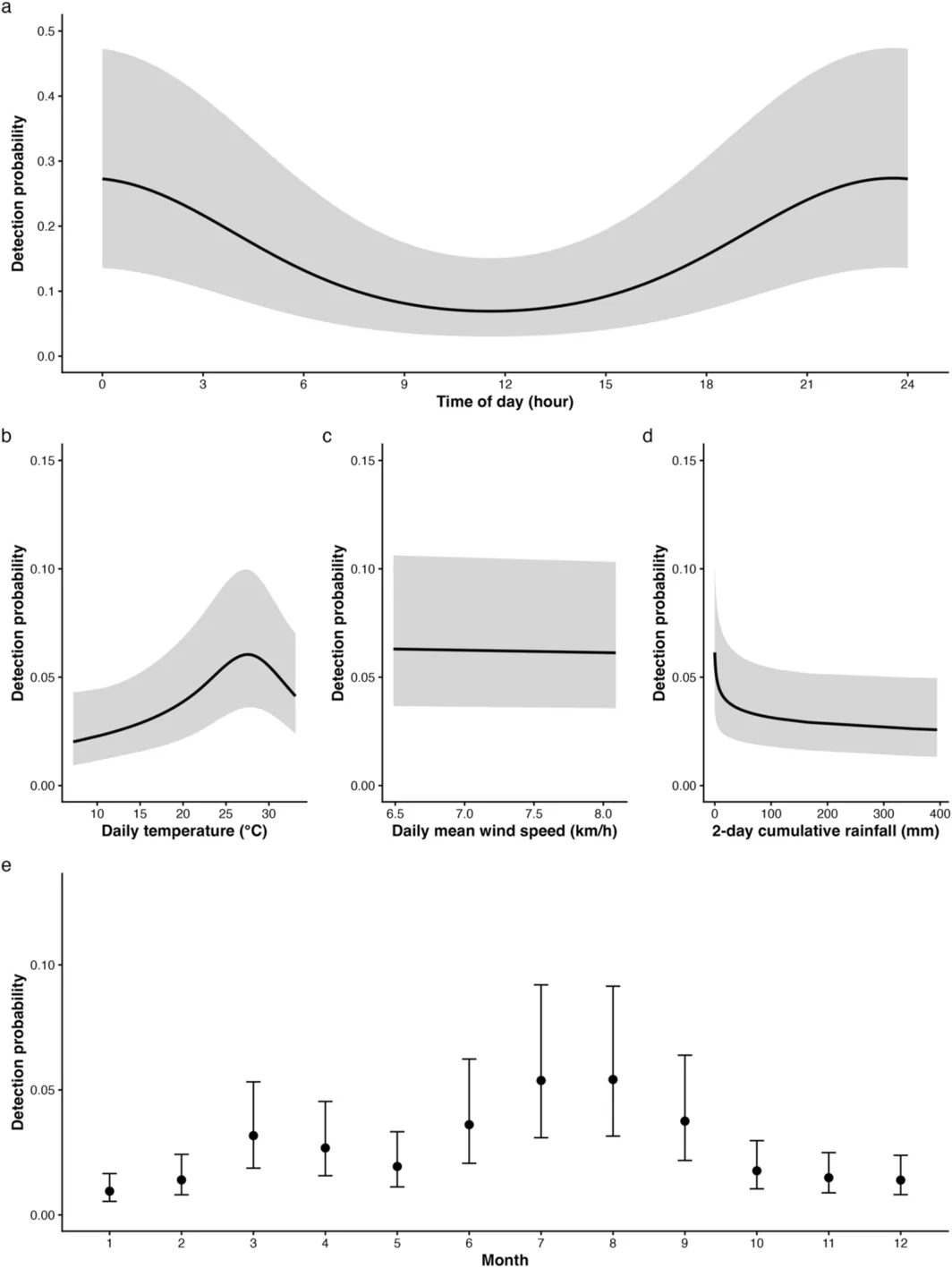
Predicted detection probability of *Liuixalus romeri* in relation to (a) time of day, (b) temperature, (c) wind speed, (d) rainfall and (e) month. Predictions were derived from generalised linear mixed models, with continuous covariates held at their mean values. Grey shading and error bars represent 95% confidence intervals.

Temperature had a significant non‐linear effect on detection probability (spline terms: *p* < 0.001 and *p* = 0.026), with a peak at around 27.5°C and declining at higher temperatures (Figure 2b). Wind speed had a mildly negative effect on detection probability (*β* = −0.090, *p* < 0.001; Figure 2c), whereas the effect of humidity was not statistically supported (*p* = 0.966). Moon phase effect was weak, with only marginal evidence for an influence of the sine component (*β =* 0.063, *p* = 0.058).

Rainfall showed an overall negative association with detection (*β* = −0.154, *p* = 0.009) (Figure 2d), but its effect varied across the diel cycle, as indicated by a significant interaction between rainfall and hour of day (interactions with sine and cosine terms: both *p* < 0.001). The additional model with terms of categorical hours showed that rainfall effects on detection were positive during early evening hours (20:00–22:00) and late night before dawn (03:00–04:00), whereas effects were negative or neutral at other times of day (Figure 3).

**FIGURE 3:**
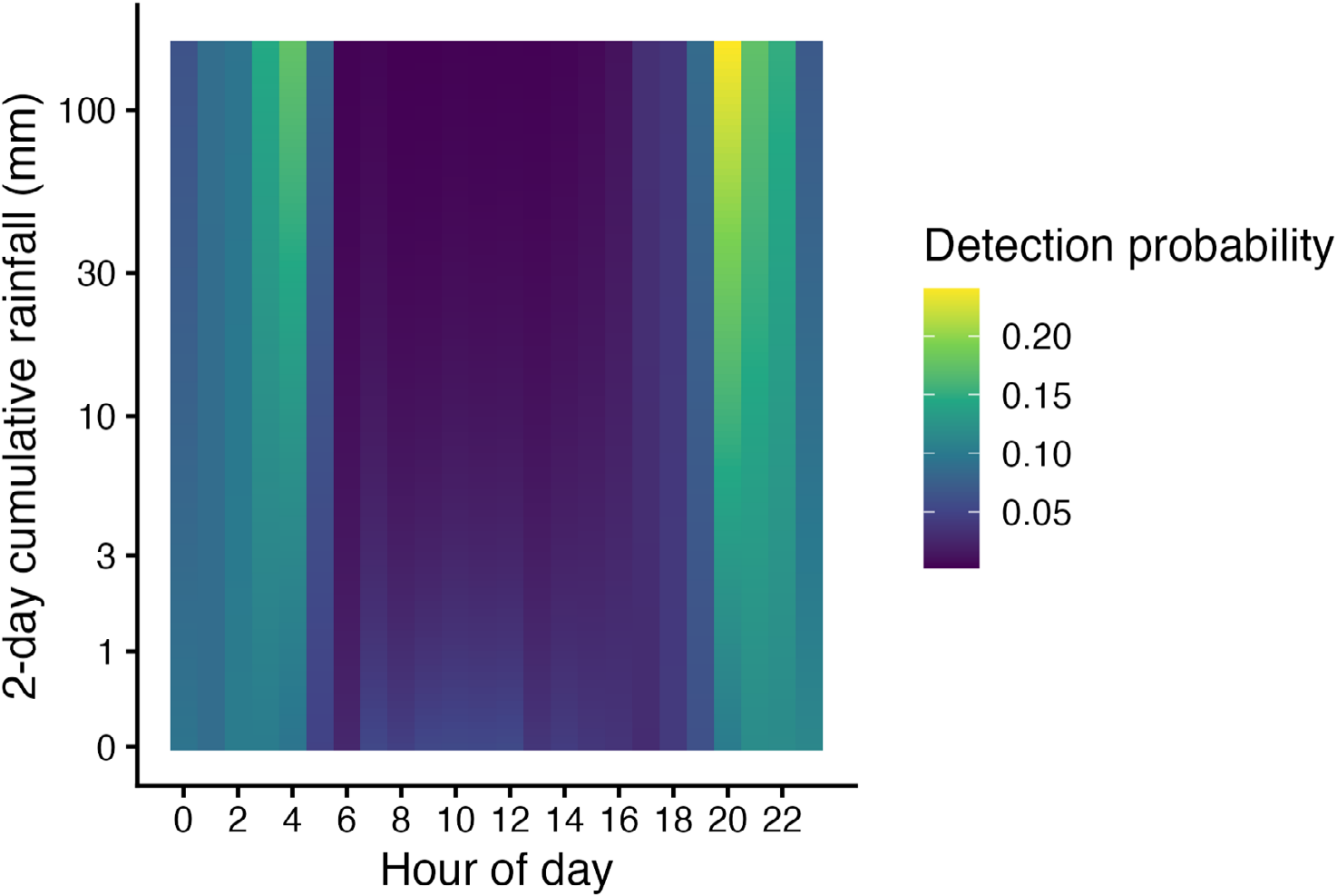
Predicted detection probability of *Liuixalus romeri* across the diel cycle and rainfall gradient. Predictions were derived from generalised linear mixed models, with continuous covariates held at their mean values and month fixed in August, corresponding to peak detection probability.

Detection probability exhibited strong seasonal variation, increasing from March and reaching a peak between June and August, before declining after September (Figure 2e). Detection probability was strongly positively autocorrelated, with the presence of calls in the previous hour substantially increasing the likelihood of detection (*β* = 2.470, *p* < 0.001).

Modelling results for calling index were consistent with the results for detection probability and are therefore presented in Appendix S2.

Concordance between PAM and active call surveys increased with the PAM sampling window (Table 2). Under exact temporal (hourly) matching, only 10 active detections were detected by PAM, with 47 active‐only detections and one PAM‐only detection (Cohen's kappa = 0.17). When PAM data were aggregated across the full night, concordant detections increased to 37, with 20 active‐only detections and 13 PAM‐only detections (Cohen's kappa = 0.47). Active call surveys detected *L. romeri* with a naïve detection probability of 0.44 (95% CI = 0.36–0.53). PAM detection probability was low during the corresponding hour (0.08, 95% CI = 0.05–0.14) but increased to 0.41 (95% CI = 0.32–0.50) when PAM data were aggregated across the full night. Detection probability from active call surveys did not differ from that of PAM when PAM data were aggregated across the full night (*p* = 0.30) but was higher than PAM detection probability during the corresponding hour (*p* < 0.001).

**TABLE 2:** Contingency table comparing detection of *Liuixalus romeri* using passive acoustic monitoring (PAM) and active call surveys in Hong Kong.

Exact hours		
	Active detected	Active not detected
PAM detected	10	1
PAM not detected	47	65
Same night		
	Active detected	Active not detected
PAM detected	37	13
PAM not detected	20	53

## 4 Discussion

In this study, we investigated how environmental factors influence detection probability using passive acoustic monitoring (PAM) for the Endangered *L. romeri* . We found that detection probability is influenced by multiple weather and temporal variables, and this information can help improve detection and estimation of abundance in manual surveys. In addition, PAM and active call surveys' concordance broadly agreed on whether *L. romeri* was detected on a given night when aggregating PAM data across the full night.

### 4.1 Diel Activity Pattern and Rainfall

Our model of *L. romeri* call detection revealed a clear diel pattern, with detection peaking during nighttime hours, consistent with predominantly nocturnal calling behaviours reported in other frog species (Callaghan and Rowley 2020). Modelling hour‐specific detection probabilities across rainfall levels revealed a bimodal distribution, with two peaks in detection during early night (20:00–22:00) and before dawn (03:00–04:00) (Figure 3). Such bimodal patterns have been documented in other species, where call detection is concentrated around dusk and dawn (Ospina et al. 2013; Van Sluys et al. 2012). The second peak before dawn may be associated with increased calling activity to defend retreat sites and reduce intraspecific competition (Stewart and Rand 1992). Overall, the diel pattern suggests that early evening is the optimal survey window for detecting *L. romeri* . Although there is concordance with studies on other frog species, pilot studies on other target species may be needed to refine and understand their activity patterns.

Rainfall influenced detection probability, but its effect varied across the diel cycle, as indicated by a significant interaction with time of day. Although the main effect of rainfall was negative, this represents an average effect across all hours and should be interpreted cautiously. Previous studies have reported both positive and negative effects of rainfall on amphibian calling activity. In some species, rainfall increases calling activity (Saenz et al. 2006; Xie et al. 2017), whereas in others, particularly small‐bodied species, heavy rainfall may reduce activity due to risks of displacement or egg loss (Heard et al. 2015; Ospina et al. 2013). In addition, rainfall can reduce detectability through acoustic masking, where rain noise interferes with call detection (Sánchez‐Giraldo et al. 2020). In *L. romeri* , detection generally decreased with increasing rainfall during most hours of the day but increased during peak calling periods (20:00–22:00 and 03:00–04:00; Figure 3). This suggests that rainfall may enhance detection during peak activity periods but reduce detectability or calling activity at other times. Although some studies have found rainfall to be a weaker determinant compared to other environmental variables (Steen et al. 2013; Thompson et al. 2022), rain may be more important for *L. romeri* . Compared to species breeding in larger water bodies or rivers, *L. romeri* relies on shallow or temporary water bodies whose availability is greatly influenced by short‐term cumulative rainfall (Leung et al. 2025; Ospina et al. 2013). Overall, surveys conducted during peak calling periods following recent rainfall are likely to maximise detection.

The use of coarser environmental variables may limit a more nuanced understanding of calling behaviour. While such variables are practical for informing survey planning, some species may respond more strongly to short‐term environmental changes, such as immediate rainfall events (Brodie et al. 2020). In this study, rainfall was quantified as a two‐day cumulative total. Although this captures broader moisture conditions, shorter‐term rainfall (e.g., within the preceding few hours) may be more influential in shaping calling activity (Chirino et al. 2025). Future studies should therefore investigate finer temporal scales of weather variables to improve survey optimisation.

### 4.2 Seasonality, Temperature and Wind

Seasonality was observed in the acoustic breeding phenology of *L. romeri* , with call detections largely restricted to March to September, with the peak between June and August. This is consistent with the species' breeding phenology (Lau 1998) and supports previous studies showing that the time of year can be the strongest determinant of frog calling activity (Thompson et al. 2022).

Temperature exhibited a non‐linear relationship with detection probability, peaking at approximately 27.5°C. This concords with studies demonstrating increased frog activity with rising temperature (Navas et al. 2008). However, excessively high temperatures may increase desiccation risk (Chirino et al. 2025). Wind speed was negatively associated with detection probability, which may reflect increased desiccation as well as sound interference on detectability (Leung et al. 2025; Oseen and Wassersug 2002).

We could not identify a specific temperature threshold to optimise survey conditions for two reasons. First, variation in temperature between days during the wet season is relatively limited compared to other variables such as rainfall and wind speed. Second, as temperature was measured at a daily scale, it may not accurately reflect microclimatic conditions during survey periods. In practice, targeting surveys within the peak season and time is likely to be more informative.

### 4.3 Application of PAM

Passive acoustic monitoring (PAM) and active call surveys showed moderate agreement (Cohen's kappa = 0.47), with 73% (90 of 123) of survey nights resulting in concordant detections or non‐detections. Similarly, active call surveys and a full night of PAM recording resulted in similar naïve detection probabilities (0.44 and 0.41). This level of agreement suggests that PAM is useful for detecting the presence of *L. romeri* , although neither method can fully replace the other (Hoefer et al. 2023). PAM detected *L. romeri* on 13 nights where active call surveys did not. These PAM‐only detections likely reflect the greater temporal coverage of continuous PAM sampling, which can capture intermittent calling activity. In contrast, active call surveys detected *L. romeri* on 20 nights where PAM did not. These active‐only detections may reflect false negatives generated by the automated classifier because a conservative detection threshold (0.3) was used in this study.

The conservative threshold prioritised precision over recall, resulting in high precision (0.89) but low recall (0.34). Consequently, a substantial proportion of true calls may not have been picked up by the classifier at this threshold. Although the validation dataset included recordings from different times of day and rainfall conditions, we estimated overall classifier performance and could not determine whether recall varied systematically with time of day or rainfall. Variations in classifier recall could bias estimates of environmental effects if, for example, calls are more difficult to detect on rainy and windier nights (lower signal‐to‐noise ratio) (Sánchez‐Giraldo et al. 2020). Thus, detectability bias caused by environmental predictors should be considered when interpreting the diel and rainfall relationships in this study. Future studies should examine whether classifier performance varies with time of day and rainfall and whether the results are consistent across different detection thresholds.

Overall, our results suggest that PAM and manual surveys are complementary. PAM is particularly suitable for long‐term monitoring of species presence, occupancy, calling phenology and changes in distribution across multiple sites because of its ability to continuously sample over extended periods (Perea et al. 2026; Ross et al. 2023). Detection performance may be further improved through expansion of the training dataset and optimisation of classifier thresholds. Manual surveys remain valuable for estimating abundance and collecting demographic information that cannot be obtained acoustically, including observations of females, tadpoles and eggs (Kaczmarski et al. 2025).

Our results also have implications for survey duration. The short recording duration used in this study (5 min) may have limited detection probability. Under optimal conditions (June–August, 20:00–22:00), a 5‐min survey yielded a detection probability of > 0.3 and the detection curve was still increasing, suggesting that longer surveys would further increase detection probability (Figure 4). Based on these findings, a longer survey duration, such as at least 10 min per site following a 5‐min acclimatisation period, would maximise detection of *L. romeri* calls. The same principle also applies to PAM, as extending the recording duration per hour beyond 5 min, where storage capacity and battery life allow, may further increase detection probability.

**FIGURE 4:**
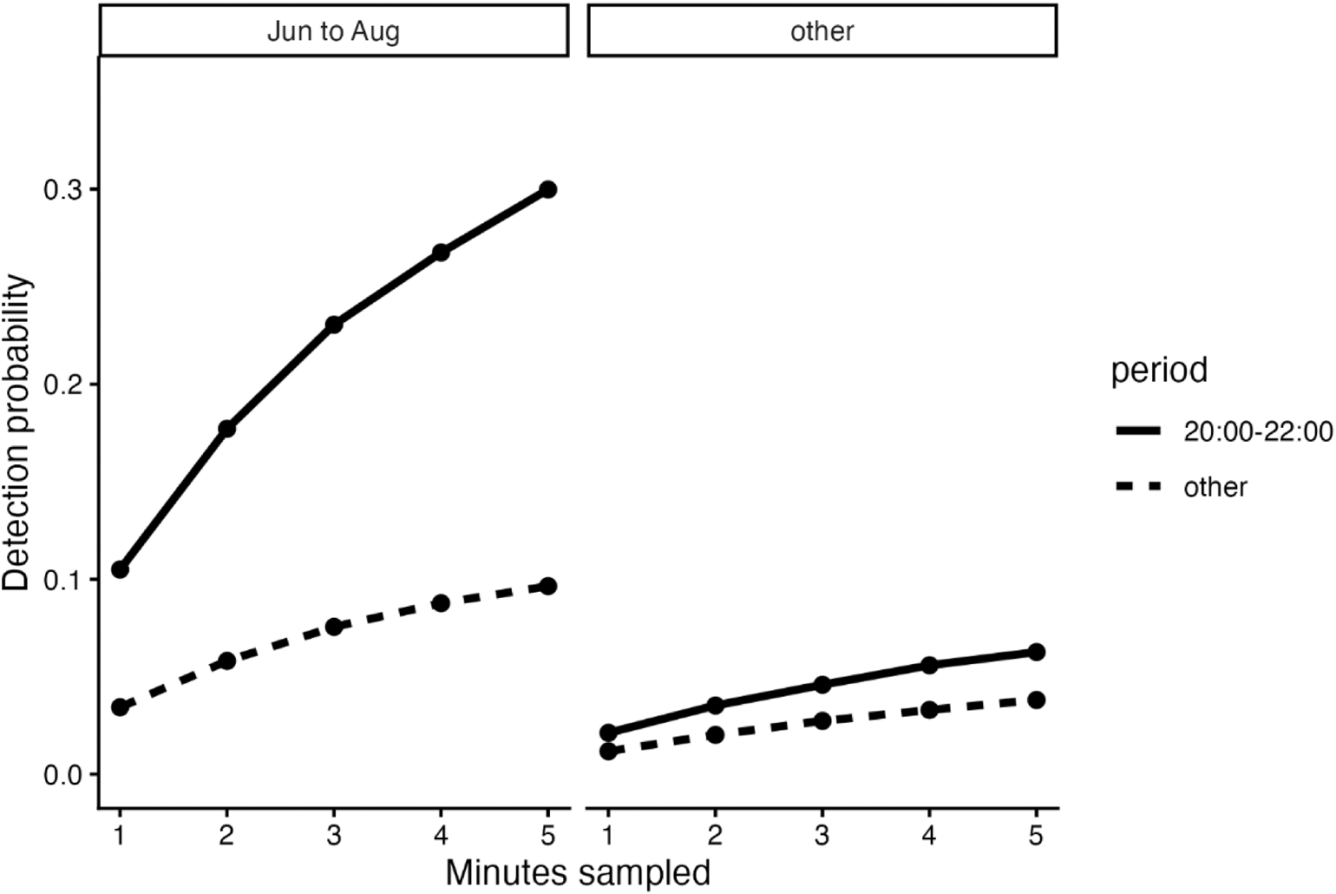
Predicted detection probability of *Liuixalus romeri* as a function of sampling effort (1–5 min), time of day and month. Predictions were derived from generalised linear mixed models, with continuous covariates held at their mean values.

## 5 Conclusions

This study demonstrates the value of PAM as a complementary tool to manual surveys. PAM cannot replace manual surveys, it provides continuous and standardised data that can inform survey optimisation. Based on our results, we recommend conducting manual surveys during the peak breeding season (June–August), particularly between 20:00 and 22:00, under conditions of low wind and with rainfall occurring on the same or preceding days. Future applications of PAM could be improved by expanding the training dataset, refining the classifier and optimising detection thresholds. Although this study focuses on a single species, similar studies should be extended to other habitats (e.g., streams, rivers and ponds) and to other amphibian species. As factors influencing calling activity vary among species (Schalk and Saenz 2015), survey protocols should be tailored to the ecology and calling behaviour of the target species.

PAM has the potential to address current conservation concerns for *L. romeri* . The introduced *E. planirostris* may affect *L. romeri* through competition for resources (Lee et al. 2016). Monitoring both species simultaneously by PAM could provide valuable information on whether the presence of *E. planirostris* may be associated with differences in *L. romeri* occurrence or calling activity, allowing comparison of *L. romeri* detection or calling phenology between introduced and unintroduced sites. Continuous recording at breeding sites could also establish standardised baselines against which reductions in calling activity following management interventions, such as mosquito larvicide application near breeding pools, could be detected. Long‐term PAM monitoring could track changes in occupancy and calling activity over time at key sites (Ross et al. 2023; Wood et al. 2019), including population strongholds and areas of conservation concern such as Chek Lap Kok, where remnant populations are suspected to be declining (Agriculture Fisheries and Conservation Department 2022). However, PAM measures acoustic detection and cannot directly estimate abundance. Therefore, sites showing changes detected by PAM should be followed by manual surveys to determine whether frog abundance has also changed.

## Author Contributions

**Yik‐Hei Sung:** conceptualization (lead), data curation (lead), formal analysis (lead), funding acquisition (lead), investigation (lead), methodology (lead), project administration (lead), resources (lead), software (lead), supervision (lead), validation (lead), visualization (lead), writing – original draft (lead), writing – review and editing (lead). **Amy Wing Lam Fok:** data curation (equal), investigation (equal), methodology (equal), writing – review and editing (equal). **Hiu Ching Yuen:** data curation (equal), investigation (equal), methodology (equal), writing – review and editing (equal). **Franco Ka Wah Leung:** conceptualization (equal), formal analysis (equal), methodology (equal), writing – review and editing (equal). **Michael Wai‐neng Lau:** conceptualization (equal), funding acquisition (equal), supervision (equal), writing – review and editing (equal). **Jia Huan Liew:** conceptualization (equal), funding acquisition (equal), writing – review and editing (equal). **Simon Yung Wa Sin:** conceptualization (equal), funding acquisition (equal), writing – review and editing (equal). **Jonathan J. Fong:** conceptualization (lead), funding acquisition (lead), project administration (lead), supervision (lead), writing – review and editing (lead).

## Funding

This work was supported by the Lantau Conservation Fund, Hong Kong SAR Government, China, RE2021‐05.

## Conflicts of Interest

The authors declare no conflicts of interest.

## Supporting information


**Appendix S1:** Fixed factors included in generalised linear mixed models (GLMMs) used to model detection probability of *Liuixalus romeri* in Hong Kong (2023–2025). One of the two recorders was excluded at each site to examine whether the results were robust to potential spatial independence. Fixed factors include diel cycle, rainfall, season (month), weather variables, moon phase and temporal autocorrelation. For season, January was used as the reference category. Significant effects (*p* < 0.05) are shown in bold.
**Appendix S2:** Fixed factors included in generalised linear mixed models (GLMMs) used to model calling index (number of 1‐min recordings with detections per hour) of Liuixalus romeri in Hong Kong (2023–2025). Fixed factors include diel cycle, rainfall, season (month), weather variables, moon phase and temporal autocorrelation. For season, January was used as the reference category. Significant effects (*p* < 0.05) are shown in bold.

## Data Availability

Data has been submitted to Figshare data repository: https://doi.org/10.6084/m9.figshare.32591685.
