# Supplementary material for: Optimising Survey Strategies for the Endangered Romer's Tree Frog (Liuixalus romeri): Effects of Diel, Seasonal and Weather Variation on Acoustic Detection
Source: Ecol Evol. 2026 Oct 14;16(10):e74491. doi: 10.1002/ece3.74491 (PMC13668485; doi:10.1002/ece3.74491)
Supplement: Supplementary file 1 [file ECE3-16-e74491-s001.docx]

Appendix

Appendix 1. Fixed factors included in generalised linear mixed models (GLMMs) used to model detection probability of *Liuixalus romeri* in Hong Kong (2023–2025). One of the two recorders was excluded at each site to examine whether the results were robust to potential spatial independence. Fixed factors include diel cycle, rainfall, season (month), weather variables, moon phase, and temporal autocorrelation. For season, January was used as the reference category. Significant effects (*p* < 0.05) are shown in bold.

| **Factor**  **category** | **Fixed factors** | **Estimate** | **SE** | ***z*** | ***p*** |
| --- | --- | --- | --- | --- | --- |
| Diel cycle | **Sine (hour)** | **-0.066** | **0.028** | **-2.375** | **0.018** |
|  | **Cosine (hour)** | **0.722** | **0.030** | **24.087** | **<0.001** |
| Rainfall | **Log (rainfall)** | **-0.131** | **0.059** | **-2.217** | **0.027** |
|  | Log (rainfall)^2^ | -0.012 | 0.014 | -0.837 | 0.403 |
| Season | February | 0.090 | 0.155 | 0.581 | 0.561 |
|  | **March** | **0.961** | **0.149** | **6.466** | **<0.001** |
|  | **April** | **0.579** | **0.190** | **3.044** | **0.002** |
|  | **May** | **0.421** | **0.193** | **2.183** | **0.029** |
|  | **June** | **1.227** | **0.214** | **5.740** | **<0.001** |
|  | **July** | **1.721** | **0.215** | **8.017** | **<0.001** |
|  | **August** | **1.782** | **0.204** | **8.741** | **<0.001** |
|  | **September** | **1.343** | **0.199** | **6.735** | **<0.001** |
|  | **October** | **0.626** | **0.170** | **3.672** | **<0.001** |
|  | **November** | **0.532** | **0.146** | **3.644** | **<0.001** |
|  | **December** | **0.391** | **0.137** | **2.862** | **<0.001** |
| Weather | **Temperature (spline 1)** | **1.810** | **0.252** | **7.185** | **<0.001** |
|  | **Temperature (spline 2)** | **3.005** | **0.761** | **3.947** | **<0.001** |
|  | **Temperature (spline 3)** | **0.672** | **0.248** | **2.715** | **0.007** |
|  | Humidity | 0.043 | 0.040 | 1.067 | 0.286 |
|  | **Wind speed** | **-0.108** | **0.025** | **-4.381** | **<0.001** |
| Moon phase | Sin (moon phase) | 0.031 | 0.033 | 0.940 | 0.347 |
|  | Cosine (moon phase) | -0.015 | 0.033 | -0.440 | 0.660 |
| Autocorrelation | **Lagged detection** | **2.381** | **0.035** | **67.998** | **<0.001** |
| Interactions | **Log (rainfall) × sine (hour)** | **-0.070** | **0.016** | **-4.387** | **<0.001** |
|  | **Log (rainfall) × cosine (hour)** | **0.225** | **0.020** | **11.257** | **<0.001** |

Appendix 2. Fixed factors included in generalised linear mixed models (GLMMs) used to model calling index (number of 1-minute recordings with detections per hour) of *Liuixalus romeri* in Hong Kong (2023–2025). Fixed factors include diel cycle, rainfall, season (month), weather variables, moon phase, and temporal autocorrelation. For season, January was used as the reference category. Significant effects (p < 0.05) are shown in bold.

| **Factor**  **category** | **Fixed factors** | **Estimate** | **SE** | ***z*** | ***p*** |
| --- | --- | --- | --- | --- | --- |
| Diel cycle | Sine (hour) | 0.008 | 0.019 | 0.445 | 0.656 |
|  | **Cosine (hour)** | **0.651** | **0.021** | **31.187** | **<0.001** |
| Rainfall | **Log** **(rainfall)** | **-0.165** | **0.057** | **-2.907** | **0.004** |
|  | Log (rainfall)^2^ | 0.004 | 0.013 | 0.277 | 0.782 |
| Season | **February** | **0.398** | **0.134** | **2.973** | **0.003** |
|  | **March** | **1.307** | **0.137** | **9.571** | **<0.001** |
|  | **April** | **1.176** | **0.177** | **6.654** | **<0.001** |
|  | **May** | **0.852** | **0.180** | **4.728** | **<0.001** |
|  | **June** | **1.521** | **0.203** | **7.509** | **<0.001** |
|  | **July** | **1.829** | **0.204** | **8.952** | **<0.001** |
|  | **August** | **1.844** | **0.194** | **9.511** | **<0.001** |
|  | **September** | **1.542** | **0.189** | **8.168** | **<0.001** |
|  | **October** | **0.747** | **0.161** | **4.651** | **<0.001** |
|  | **November** | **0.584** | **0.136** | **4.289** | **<0.001** |
|  | **December** | **0.452** | **0.125** | **3.617** | **<0.001** |
| Weather | **Temperature (spline 1)** | **1.280** | **0.213** | **6.022** | **<0.001** |
|  | **Temperature (spline 2)** | **1.675** | **0.609** | **2.750** | **0.006** |
|  | **Temperature (spline 3)** | **0.545** | **0.234** | **2.326** | **0.020** |
|  | Humidity | 0.025 | 0.037 | 0.680 | 0.496 |
|  | **Wind speed** | **-0.089** | **0.024** | **-3.774** | **<0.001** |
| Moon phase | Sin (moon phase) | 0.077 | 0.032 | 2.412 | 0.016 |
|  | Cosine (moon phase) | 0.013 | 0.032 | 0.419 | 0.675 |
| Autocorrelation | **Lagged detection** | **0.635** | **0.008** | **78.292** | **<0.001** |
| Interactions | **Log (rainfall) × sine (hour)** | **-0.045** | **0.011** | **-4.292** | **<0.001** |
|  | **Log (rainfall) × cosine (hour)** | **0.211** | **0.013** | **16.125** | **<0.001** |
